# Prediction of regional lymph node metastasis in intrahepatic cholangiocarcinoma: it’s not all about size

**DOI:** 10.1007/s00261-023-03991-1

**Published:** 2023-06-24

**Authors:** Johannes Kolck, Timo Alexander Auer, Thula Walter-Rittel, Clarissa Hosse, Aboelyazid Elkilany, Adrian Alexander Marth, Uwe Pelzer, Raphael Mohr, Felix Krenzien, Georg Lurje, Wenzel Schöning, Bernd Hamm, Dominik Geisel, Uli Fehrenbach

**Affiliations:** 1 Department of Radiology, Charité – Universitätsmedizin Berlin, Berlin, Germany; 2 BIH Biomedical Innovation Academy, Berlin Institute of Health at Charité –Universitätsmedizin Berlin, Berlin, Germany; 3 Department of Hematology/Oncology, Charité – Universitätsmedizin Berlin, Berlin, Germany; 4 Department of Hepatology and Gastroenterology, Charité – Universitätsmedizin Berlin, Berlin, Germany; 5 Department of Surgery CCM/CVK, Charité – Universitätsmedizin Berlin, Berlin, Germany

**Keywords:** Lymph node metastasis, Intrahepatic cholangiocarcinoma, Lymph node short axis, Presurgical imaging, Computed tomography

## Abstract

**Objectives:**

Lymph node metastases (LNM) are frequent in patients with intrahepatic cholangiocarcinoma (iCC) and worsen their prognosis even after surgery. Our aim was to investigate the predictive value of lymph node (LN) short axis, the most common discriminator for identifying LNM in tumor-imaging and to develop a predictive model for regional LNM in iCC taking computed tomography (CT) features of extranodal disease into account.

**Materials and methods:**

We enrolled 102 patients with pathologically proven iCC who underwent CT prior to hepatic resection and hilar lymph node dissection (LND) from 2005 to 2021. Two blinded radiologists assessed various imaging characteristics and LN diameters, which were analyzed by bivariate and multivariate logistic regression to develop a prediction model for LNM.

**Results:**

Prevalence of LNM was high (42.4 %) and estimated survival was shorter in LN-positive patients (*p* = 0.07). An LN short axis diameter of ≥ 9 mm demonstrated the highest predictive power for LNM. Three additional, statistically significant imaging features, presence of intrahepatic metastasis (*p* = 0.003), hilar tumor infiltration (*p* = 0.003), and tumor growth along the liver capsule (*p* = 0.004), were integrated into a prediction model, which substantially outperformed use of LN axis alone in ROC analysis (AUC 0.856 vs 0.701).

**Conclusions:**

LN diameter alone proved to be a relevant but unreliable imaging-marker for LNM prediction in iCC. Our proposed prognostic model, which additionally considers intrahepatic metastases and hilar and capsular infiltration, significantly improves discriminatory power. Hilar and capsular involvement might indicate direct tumor extension to lymphatic liver structures.

## Introduction

Intrahepatic cholangiocarcinoma (iCC) is a rare, but aggressive type of tumor that commonly arises from second-order bile ducts [1, 2]. Currently, iCC accounts for about 10%–12% of all malignant liver tumors [3] with an age peak between 50 and 70 years [4]. Its occurrence is associated with geographical risk factors [5], such as parasitic infection (Opisthorchis viverrini and Clonorchis sinensis) in Southeast Asia and primary sclerosing cholangitis (PSC) in the western world [6, 7]. Due to the lack of symptoms, iCCs are commonly detected at an advanced stage. Up to 54% of patients are diagnosed with unresectable tumors, and only about 35% are candidates for surgical resection [8, 9], which remains the mainstay of curatively intended therapies [10]. Despite growing knowledge and recent advances in perioperative care and surgical techniques the prognosis of patients with iCC is generally poor with a five-year survival rate below 30% [11, 12].

In various studies, lymph node metastasis (LNM) was found to have a significant impact on long-term outcomes, with a reported median overall survival (OS) of 7 to 14 months [13–17]. The 8th edition of the American Joint Committee on Cancer staging system recommends the harvest of at least six lymph nodes (LNs) to ensure adequate pN staging [18]. A possible impact on OS of other features, such as location and number of metastatic LNs, as well as the total number of LNs removed, is still under debate [17, 19, 20]. Even though presurgical imaging is routinely performed and known to be of paramount importance for initiating appropriate therapy [21–23], accurate prediction of LNM and therefore adequate extent of lymph node dissection remains difficult [24]. Since LNM has a negative impact on prognosis even after complete surgical removal, it would be desirable to diagnose LNM more reliably before patients are operated on in order to avoid excessive surgery with its corresponding morbidity and mortality in high-risk patients [25].

The objective of the present study was to investigate the predictive value of LN short axis length in iCC, a widely recognized discriminator for LNM in tumor-imaging, and to develop an improved predictive model for LNM based on additional extranodal CT imaging features.

## Materials and methods

### Patient population and image acquisition

The study was approved by the Ethics Committee prior to implementation (Internal registration number: EA2/016/14). We retrospectively enrolled 102 patients with pathologically proven mass-forming iCC who underwent multiphase CT imaging prior to liver resection including hilar lymphadenectomy from 2005 to 2021. The study population consisted of 46 women and 56 men. Average patient age at the time of surgery was 64 ± 11 years, ranging from 32 to 94 years. Excluded from the study were patients with primary lesions classified as greater than T3 according to the 2017 AJCC staging system, constellations of bilobar tumor manifestations precluding hemi hepatectomy or trisectionectomy, lymph node metastases beyond the hilar region and other extrahepatic, distant metastases (M1) on preoperative imaging. Other exclusion criteria were inadequate imaging studies, mixed histology (iCC/HCC), and primary lesions measuring < 1 cm in diameter. For each patient, we recorded the following additional outcome data: time of tumor recurrence, last follow-up, and death, if available. From the pathological reports we extracted the tumor grade, classified from G1 to G3 (well differentiated, moderately differentiated, and poorly differentiated) as well as the presence and number of LNMs.

CT examinations in the study patients were performed on different scanners and using slightly varying contrast-enhanced acquisition protocols, as 62% of preoperative CT scans were performed at external facilities and only 38% at our university hospital. Multidetector CT scanner models ranged from 16 to 64 slices. Eighty-seven CT datasets included an arterial (15–20 seconds post injection), a portal venous (35–40 seconds p.i.), and a venous phase (70–80 seconds p.i.); 12 examinations only comprised an arterial and a venous contrast phase. An additional delayed venous phase (100–120 seconds p.i.) was acquired in only 3 CT examinations. All scans were available for assessment in our department’s picture archiving and communication system (PACS).

### Image analysis

For our retrospective analysis, the presurgical CT scans were assessed on dedicated PACS workstations (Centricity PACS, GE Healthcare, Barrington, IL) by two radiologists: reader 1, TA, and reader 2, JK, with 6 and 4 years of experience in abdominal imaging. The readers were aware of the pathologically proven diagnosis of iCC, but otherwise blinded to all clinical data, especially the patients’ lymph node status. In consensus, both readers defined the intrahepatic index lesion as the largest lesion in the axial plane. The images were reviewed for the following qualitative and quantitative features: maximum size of the index lesion (in mm) in axial plane, lesion shape classified as round, lobulated, or infiltrative. Lesion attenuation in venous phase (70–80 seconds p.i.) was categorized as homogenous or heterogenous. Tumor necrosis, defined as the absence of contrast enhancement in hypodense tumor areas across all contrast phases; tumor growth within 5 mm of the hilar region, contiguity with the liver capsule outside the hilar region, defined as tumor extension with direct capsular contact over a distance greater than 5 mm, and its extent into the individual segments were noted. Liver tumors infiltrating the hilar region were only assigned the attribute of hilar infiltration. In case of additional capsule contact outside the hilar region, these tumors were also scored for that attribute. If present, we reported encasement of the central portal vein, defined as more than 180° of circumferential tumor contact to the right or left intrahepatic portal vein, and involvement of the biliary system in terms of biliary obstruction. On contrast-enhanced images, readers evaluated the presence of perilesional perfusion abnormalities and the presence or absence of arterial rim enhancement. Moreover, we measured minimum, mean, and maximum Hounsfield units (HU) in the lesion centre and mean HU in an area of unaffected liver parenchyma. For all measurements the region of interest (ROI) had a minimum diameter of 1.5 cm. Intrahepatic metastatic lesions, if present, were counted and classified as local, or distant lesions. Distant metastases were defined as located outside the liver segment of the index lesion. The distance between the most distant metastasis and the index lesion was measured. The maximum spleen size in axial plane (anteroposterior) was measured as a potential correlate of portal hypertension. To assess LN status, we recorded the presence of hilar LNs and measured the maximum short axis diameter in millimetres. As previous studies have highlighted the low precision of preoperative imaging in assessing LN status [26], we used a rather low cut-off value for the short axis diameter to identify metastatic LNs. In contrast to the RECIST guidelines, which define LNs with short axes ≥ 15 mm as target lesions, the two readers recorded all nodes with a short axis diameter of greater than 5 mm [27]. The number of potentially metastatic LN was categorized in three groups: (1) less than five, (2) five to ten, and (3) more than ten. The presence or absence of enlarged supradiaphragmatic LNs was noted.

### Statistical analysis

Descriptive statistics for all numeric and categorical variables were calculated as median with interquartile range (IQR) and frequency (%), respectively. Overall survival was defined as the period between surgery for iCC and the time of last follow-up or death from any cause. The Kaplan–Meier method was used to assess the differences in survival between patients with and without LNM.

We implemented a logistic regression analysis to assess the relationship of preoperatively available variables with LNM and pathological tumor grade. AJCC stages were omitted for statistical analysis, because patients with primary lesions > T3 or distant metastases (M1) were excluded from the study and criteria for T stages (T1a to T3) namely lesion size, vascular infiltration, and intrahepatic metastasis, were included as individual parameters in the calculations. All variables that were significant in the bivariate analysis (*p* < 0.05) were included in the multivariate logistic regression model. Receiver operating characteristic (ROC) analysis was conducted to calculate the area under the curve (AUC) for both LN short axis and the logistic regression model. The Youden-index was calculated as potential cut-off for LN short axis diameter. Statistical significance was assumed for all *p*-values < 0.05. Analyses were performed with SPSS, v27 (IBM Corp. Armonk, NY, USA) and Stata, v14 (StataCorp LLC, College Station, Texas, USA).

## Results

### General characteristics of the study population

**Fig. 1:**
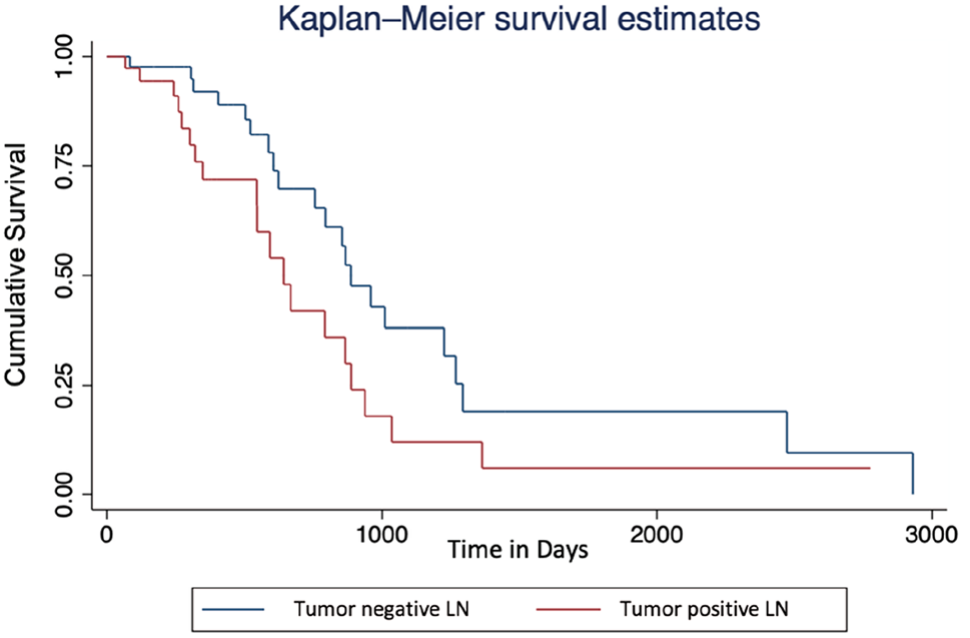
Kaplan–Meier curves of overall survival comparing positive (*n* = 42) and negative lymph node (LN) status (*n* = 57) of patients with intrahepatic cholangiocarcinoma. The histologically proven presence of lymph node metastases (LNM) almost significantly shortened patient survival (*p* = 0.07)

The final patient population we analysed consisted of 102 patients (Table 1). Median age on the day of surgery was 64 years (IQR 57.3–72.0). Slightly more than half of the patients were male (*n* = 56; 55%), the smaller proportion was female (*n* = 46; 45%). Based on histopathological reports, there were 42 cases with tumor-positive, 57 with tumor-negative, and 3 with inconclusive or missing LN status. The majority of lesions were graded as moderately differentiated (G2, *n* = 70), while well differentiated (G1, *n* = 6) and poorly differentiated (G3, *n* = 21) lesions were far less common. In five reports, tumor grading was missing or inconclusive. Complete follow-up was available for 65.7% of patients (*n* = 67), as a number of patients were admitted from external centres and were lost to follow-up. Median time to recurrence was 211.5 days (IQR 96–461.8). Survival of patients with LNM was shorter than in patients with nonmetastatic LNs (*p* = 0.007), consistent with the results of previous studies [26, 28, 29] (Figure 1).

**Table 1:** Overview of patient collective (age and gender distribution), lymph node (LN) status and recurrence

Patient collective (*n* = 102)	Demographics	Female	46
		Male	56
		Mean age	64 ± 11
	LN status	Positive	42
		Negative	57
		*Unknown*	*3*
	Recurrence	Yes	58
		No	8
		*Unknown*	*36*

### Imaging features

The median size of the index lesion was 73.5 (IQR 53.3–91.5) mm. The majority of index lesions were characterized as heterogeneous (*n* = 66; 64.7%) and showed a lobulated shape (*n* = 80; 78.4 %), while round (*n* = 13; 12.8%) and infiltrative growth patterns (*n* = 9; 8.8%) were identified far less commonly. Intratumoral necrosis was observed in 62 cases (60.8%), central encasement of the portal vein in 29 (28.4%), and biliary obstruction in 55 patients (53.9%). The majority of index lesions exhibited tumor growth along the liver capsule (*n* = 75; 73.4 %) and were localized in the right lobe (55.6 %), whereas a minority showed spreading to the hilar area (*n* = 38; 37.3%). The least affected liver segment was the caudate lobe (*n* = 3; 2.9%). Median spleen size in the axial plane was 107 mm (IQR 96.5–120 mm). In terms of contrast enhancement, a little less than half of the index lesions showed arterial rim enhancement (*n* = 47; 46.1%), an even smaller proportion presented with perilesional perfusion abnormalities (*n* = 31; 30.4%). The medians of mean, minimum and maximum lesion HU were 58 (IQR 47–74.5), − 22 (IQR − 51.5 to − 0.5), and 148 (IQR 117.3–177.8), respectively. Median mean HU in unaffected liver parenchyma was 105 (IQR 92.5–114.8). Intrahepatic metastases were observed in 47 % of cases (*n* = 48), with a median of 3 lesions (IQR 1–5). Out of the tumors with intrahepatic metastasis, 79.1 % (*n* = 38) presented distant hepatic satellites, i.e., metastases outside the liver segment harbouring the index lesion.

### Assessment of lymph node status

Hilar lymph node dissection (LND) of at least 6 LNs was performed in all patients. Three patients in the final study population had inconclusive or missing pathological reports on LNM. In the remaining group of 99 patients, pathologically proven nodal metastasis was observed in 42.4 % of cases (*n* = 42). The median number of metastatic LNs was 2 (IQR 1–3.5). The majority of patients had a LN status with short axis diameter ≥ 5 mm (*n* = 88; 86%), which we set as threshold for our analysis. The main share of patients with LN status ≥ 5 mm had more than 5 but less than 10 lymph nodes meeting this criterion (*n* = 48; 54.6%). Supradiaphragmatic enlarged LNs were observed in 42 cases (41.2%) with a median short axis diameter of 10 mm (IQR 8–12.3 mm). At ≥ 5 mm, specificity for LNM detection was poor with 24.6 %, while sensitivity was 100%. Conversely, a threshold of 16 mm would have resulted in high specificity (90.7%) but low sensitivity of 21.4% with a high proportion of false negative predictions. The Youden-index was calculated for all potential cut-off values; the highest score was reached at a threshold of ≥ 9 mm (Table 2). For this threshold, radiologic-pathologic LN status was inconsistent in 32.3% of cases (*n* = 32).

**Table 2:** Detailed report of the sensitivity and specificity of the lymph node (LN) short axis

LN short axis: detailed report of sensitivity and specificity					
Cut-point (mm)	Sensitivity	Specificity (%)	LR+	LR−	Youden-index
>= 6	100.00%	0.00	1		0
>= 7	95.24%	4.65	0.9988	1.0238	− 0.0011
>= 8	92.86%	20.93	1.1744	0.3413	0.1379
>= 9	85.71%	39.53	1.4176	0.3613	0.2524*
>= 10	76.19%	48.84	1.4892	0.4875	0.2503
>= 11	59.52%	60.47	1.5056	0.6694	0.1999
>= 12	45.24%	74.42	1.7684	0.7359	0.1966
>= 13	33.33%	83.72	2.0476	0.7963	0.1705
>= 14	26.19%	86.05	1.877	0.8578	0.1224
>= 15	21.43%	88.37	1.8429	0.8891	0.098
>= 16	21.43%	90.70	2.3036	0.8663	0.1213
>= 17	16.67%	93.02	2.3889	0.8958	0.0969
>= 18	11.90%	93.02	1.7063	0.947	0.0492
>= 19	9.52%	95.35	2.0476	0.9489	0.0487
>= 20	9.52%	97.67	4.0952	0.9263	0.0719
>= 22	7.14%	100.00		0.9286	0.0714
>= 30	2.38%	100.00		0.9762	0.0238
> 30	0	100.00		1	0

Adjusting the threshold for LN diameter inevitably leads to a degradation of either sensitivity or specificity. Highest Youden-index is marked with*

### Prediction model for LNM

All qualitative and quantitative imaging features we investigated were assessed in correlation with histologically proven LNM in a bivariate analysis. Lesion size in mm (CI 1.002–1.032; *p* = 0.022), tumor necrosis (CI 1.375–7.988; *p* = 0.008), infiltration of liver segment VI (CI 1.146–8.937; *p* = 0.026) and the hilar area (CI 1.027–5.393; *p* = 0.043), growth along the liver capsule (CI 1.733–17.719; *p* = 0.004), and a LN short axis diameter greater than 9 mm (CI 2.607–19.627; *p* < 0.001), presence of intrahepatic metastasis in general (CI 1.851–10.144; *p* = 0.001) as well as the discriminated presence of local (CI 1.565–10.222; *p* = 0.004) and distant intrahepatic metastasis (CI 1.110–5.915; *p* = 0.027) were each associated with higher odds for LNM. Aiming to maintain simplicity and consistency by representing each factor with a single variable, we summarized local and distant metastasis to presence of intrahepatic metastasis in the multivariable model, (Table 3).

**Table 3:** Imaging features in bivariate logistic regression

Bivariate logistic regression				
	Odds ratio	95% C.I.for EXP(B)		Sig.
		Lower	Upper	
Appearance	1.509	0.651	3.500	0.338
Arterial rim enhancement	0.663	0.294	1.497	0.322
Bile obstruction	1.200	0.538	2.677	0.656
Capsular contact	5.542	1.733	17.719	**0.004**
Encasement of portal vein	0.769	0.317	1.865	0.561
Infiltration of hilar area	2.353	1.027	5.393	**0.043**
LN short axis (≥ 9 mm)	7.154	2.607	19.627	**0.000**
Mean lesion HU	0.955	0.978	1.013	0.577
Metastasis	4.333	1.851	10.144	**0.001**
Necrosis	3.314	1.375	7.988	**0.008**
Perfusion abnormality	1.176	0.500	2.770	0.710
Segment I	2.800	0.245	31.949	0.407
Segment II	1.201	0.476	3.029	0.698
Segment III	1.484	0.573	3.844	0.416
Segment IVa	1.904	0.812	4.467	0.139
Segment IVb	3.202	1.147	8.937	**0.026**
Segment V	1.700	0.730	3.962	0.219
Segment VI	1.850	0.820	4.173	0.138
Segment VII	2.133	0.803	5.669	0.129
Segment VIII	0.710	0.294	1.712	0.446
Shape	1.140	0.487	2.669	0.763
Size (in mm)	1.017	1.002	1.032	**0.022**
Spleen size	1.007	0.986	1.028	0.535

Statistically significant values are printed in bold

**Fig. 2:**
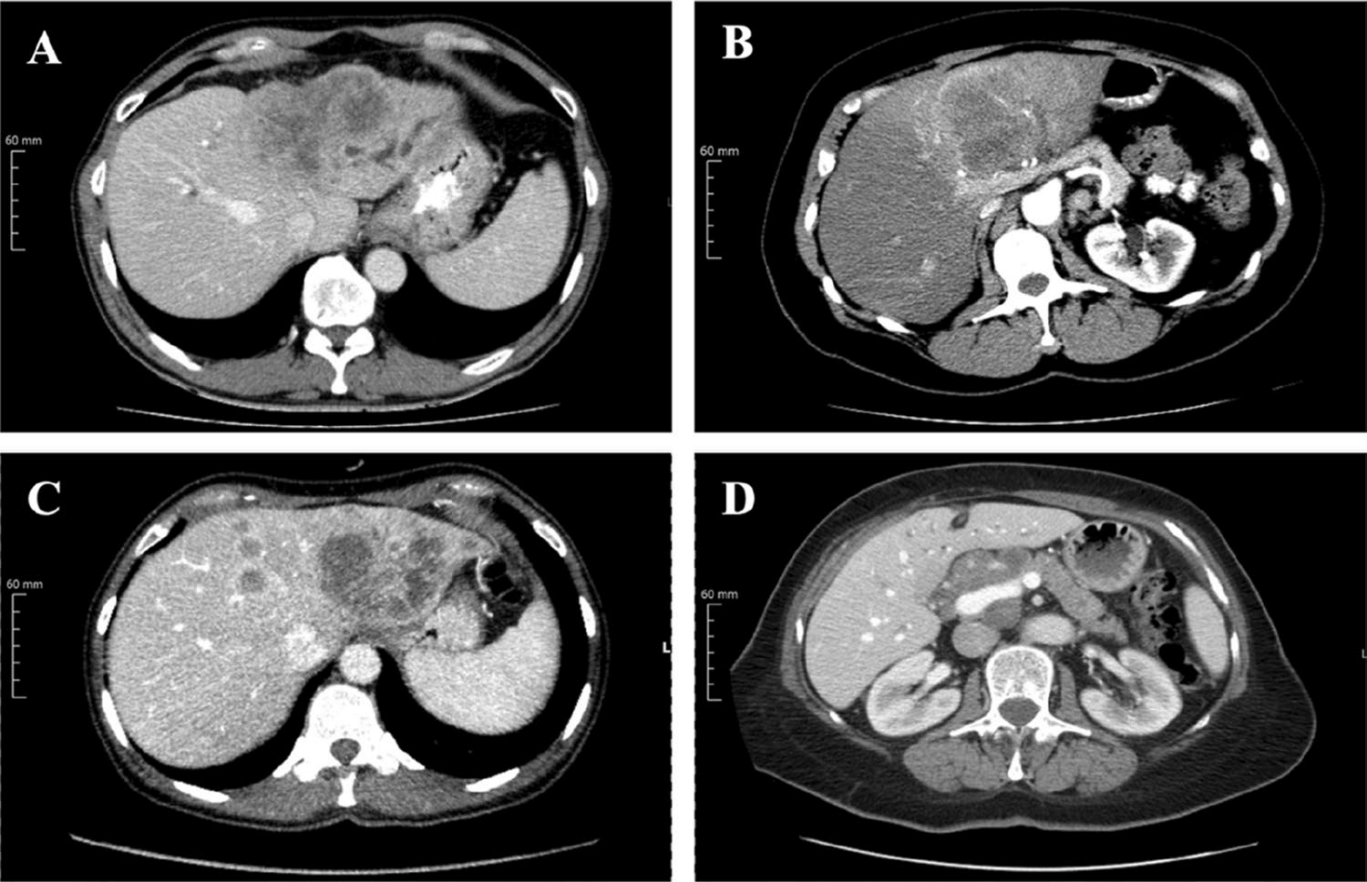
Depicted are the four statistically significant imaging features upon which the predictive model was built: capsular contact of the tumor, here in the left lobe (**a**). Infiltration of the hilar area, in this case with spread adjacent to the portal vein and hepatic artery branches (**b**). Intrahepatic metastasis, with the main lesion located in the left lobe—later undergoing trisectorectomy (**c**). Enlarged lymph nodes in the liver hilum (**d**)

**Fig. 3:**
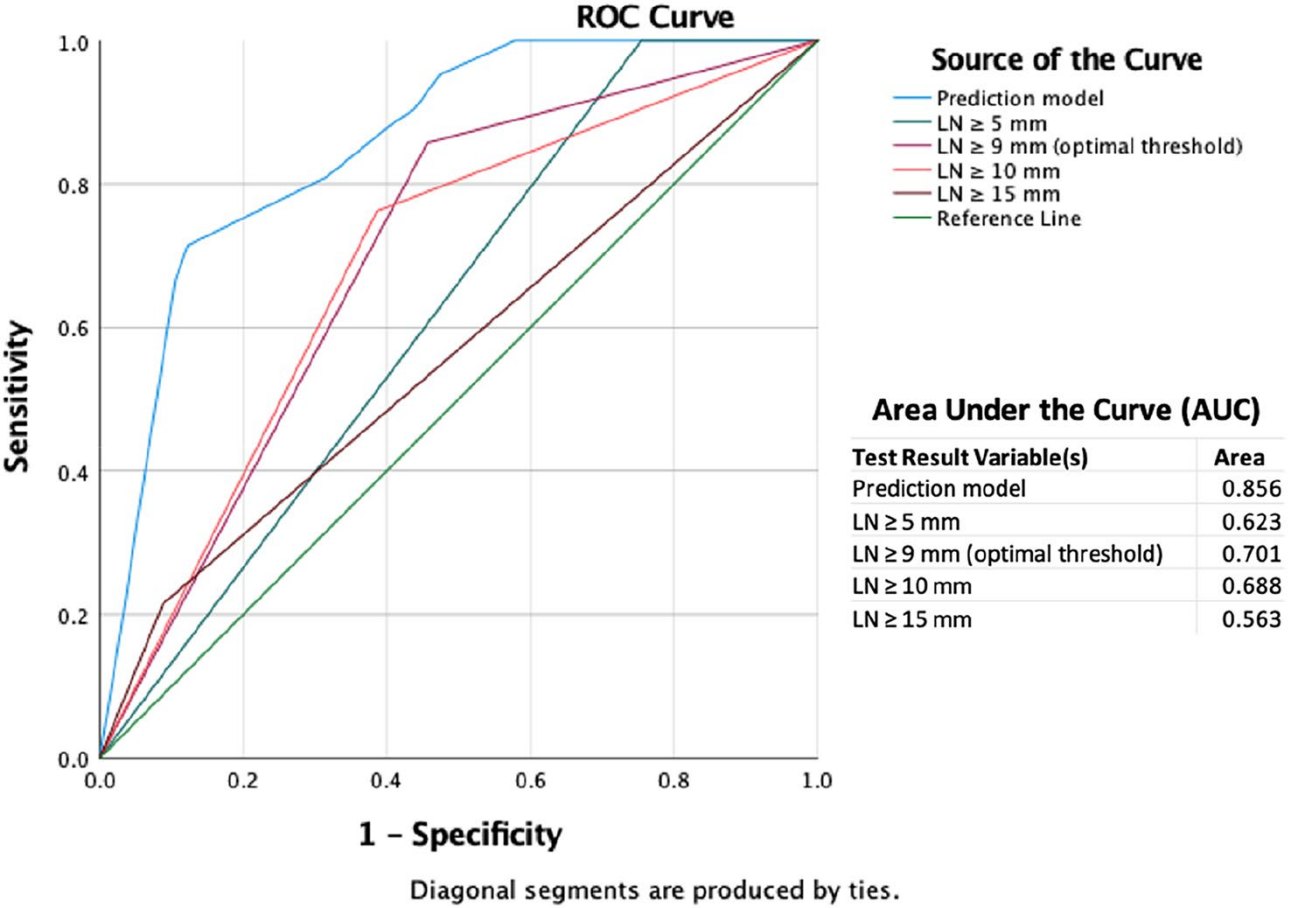
Receiver operating curves of lymph node (LN) diameter thresholds, ≥ 5 mm, ≥ 9 mm, ≥ 10 mm, ≥ 15 mm and the prediction model based on four imaging features. In predicting lymph node metastases, the model outperformed all thresholds for short LN axes

Only four variables continued to show significance in the multivariable logistic regression model: LN diameter ≥ 9 mm (CI 2.787–32.475; *p* < 0.001), presence of intrahepatic metastasis (CI 1.805–16.313; *p* = 0.003), hilar infiltration (CI 1.855–22.224; *p* = 0.003), and tumor growth along the liver capsule (CI 1.893–31.400; *p* = 0.004) were statistically significant (Table 4 and Fig. 2). The model considered all of the four variables simultaneously and demonstrated an accuracy of 80.8% in prediction of the correct LN status. Tumor-positive LNs were correctly identified in 87.7% (50/57 of cases), tumor-negative LNs in 71.4% (30/42 patients). ROC analysis of the model yielded an AUC of 0.856, exceeding the predictive power of ≥ 5 mm, ≥ 9 mm, ≥ 10 mm, and ≥ 15 mm LN short axis diameters alone, which yielded AUCs of 0.623, 0.701, 0.688, and 0.563, respectively (Fig. 3).

**Table 4:** Statistically significant imaging features that were incorporated in to the prediction model, which substantially outperformed the predictive power of lymph node (LN) short axis alone

Multivariate logistic regression				
	Odds ratio	95% C.I.for EXP(B)		Sig.
		Lower	Upper	
Infiltration of hilus area	6.420	1.855	22.224	0.003
Capsular contact	7.710	1.893	31.400	0.004
Metastasis	5.426	1.805	16.313	0.003
LN short axis (≥ 9 mm)	9.514	2.787	32.475	0.000

## Discussion

ICC is a comparatively rare but aggressive liver tumor. Even after curatively intended liver resection, which is only feasible in 20%–35% of cases [9, 10], patients have a dismal five-year survival rate of only 30% [11, 12]. Presence of lymph node metastasis has been identified as an important prognostic factor after surgery, further worsening OS to 7–14 months [13–17].

In this study, we aimed to identify imaging features that improve the detection of LNM in presurgical imaging of patients with intrahepatic cholangiocarcinoma (iCC). In terms of size, we identified ≥ 9 mm as the most appropriate short axis diameter for LNM detection.

However, the radiologic-pathologic inconsistency for this cut-off remained unconvincing at 32.3% and its sole consideration for LNM prediction appeared insufficient. Therefore, we developed a prediction model based on four significant imaging features. Our model outperformed the assessment of lymph node diameter alone, achieving an AUC of 0.856 compared to 0.701 and correctly predicted the lymph node status in 80.8% of cases. In addition to LN diameter ≥ 9 mm (CI 2.787–32.475; *p* < 0.001), the model simultaneously included the presence of intrahepatic metastasis (CI 1.805–16.313; *p* = 0.003), hilar infiltration (CI 1.855–22.224; *p* = 0.003) and tumor growth along the liver capsule outside the hilar region (CI 1.893–31.400; *p* = 0.004).

A major advantage of the study population we analysed is that LN status was histopathologically verified by surgical specimens. Overall incidence of nodal metastasis was 42.4%, which is high compared to data reported in previous studies [30]. This discrepancy might be attributed to the routinely performed LND in all study patients, which increased the likelihood of LNM harvest, even in patients with inconspicuous LN short axis diameters on presurgical imaging. Enlarged short axis diameters of LNs is a widely accepted indicator for LNM in cancer imaging [27, 31, 32], also representing an important pillar of preoperative diagnostics in iCC [31, 33]. However, there are various underlying conditions that may cause portal lymphadenopathy and thus interfere with LNM identification based only on size in iCC imaging [34]. Two commonly observed conditions that cause false positive results are for example impaired biliary drainage and cholangitis. On the other hand, the accepted threshold for RECIST target lesions, LN diameter ≥ 15 mm, appears to be too generous. In our study collective that cut-off yielded limited discriminatory power with an AUC of only 0.563.

Regarding the other discriminators, the presence of intrahepatic metastasis has been consistently associated with lower recurrence-free survival, as reported by previous studies [22, 35]. Infiltration into the hilar region was previously linked to LNM by at least two study groups [36, 37], while the relationship between lesion growth along the liver capsule and lymph node metastasis has received less attention in the literature. However, both features, capsule contact und hilar infiltration might indicate direct infiltration of the perihepatic lymphatic system. Studies investigating the liver’s lymphatic drainage identified a capsular or superficial and a deep lymphatic system [38]. The deep system drains the main share of lymph from the liver, propagating it from the space of Disse, to the space of Mall, to lymphatic vessels along the portal triad to hilar nodes, thereby respecting segmental anatomy. In addition, direct drainage from the liver parenchyma was observed not only to hilar but also to thoracic lymph nodes [39]. Thus, tumor extending to the hilar area, where segmental lymph drainages converge, and along the liver capsule, which appears to have its own lymphatic drainage system, might enhance spread of tumor cells or indicate direct infiltration of lymphatic structures. In the future, a better understanding of drainage patterns to hilar and thoracic LNs might help in more accurately guiding surgical (LND) and oncological decision making.

Our study has some limitations. Due to the retrospective design, a selection bias is unavoidable. Patients with more aggressive iCC might be underrepresented, as curatively intended resection is less often feasible in these cases. The generalizability and validity of our results are limited due to the moderate size of our single-centre study population of 102 patients. Due to a considerable number of patients lost to follow-up, our study has limited statistical power to evaluate the long-term outcomes of patients. Moreover, the true accuracy of LNM is likely dependent on the extent of LND. Even though LND was, in contrast to many other studies, performed in all enrolled patients, the number of resected LN is often dependent on individual decisions of the surgeon. The highly variable number of metastatic LNs [1–10] might indicate a hidden proportion of LNMs. Approaches to overcome these diagnostic uncertainties might be the use of 18F-flourideoxyglucose positron emission tomography (18-F-FDG-PET) in critical cases [40] and of even more standardized surgical procedures. In addition, the CT studies analysed here were performed by multiple institutions using slightly varying protocols for contrast-enhanced series. This limits the diagnostic value of some quantitative imaging features, so that our analysis focused on qualitative features, which are less dependent on scanner technique.

In conclusion, LNM is very common in patients suffering from iCC and worsens their prognosis even after surgery and detection of metastatic LNs in presurgical imaging remains a challenge. In our study population, analysis of short axis LN diameters revealed ≥ 9 mm as the optimal cut-off. Nevertheless, reliance on LN short axis diameter alone resulted in low diagnostic quality. Discriminatory power was improved by our proposed LNM prediction model, which incorporates four imaging features: presence of hepatic metastasis, LN short axis ≥ 9 mm, infiltration of hilus area, and tumor growth along the liver capsule.

## Abbreviations

AUC: Area under the curve
CI: Confidence interval
CT: Computed tomography
HCC: Hepatocellular carcinoma
HU: Hounsfield units
iCC: Intrahepatic cholangiocarcinoma
IQR: Interquartile range
ROC: Receiver operating characteristic
LN: Lymph node
LND: Lymph node dissection
LNM: Lymph node metastasis
OS: Overall survival
PACS: Picture archiving and communication system
PSC: Primary sclerosing cholangitis
ROI: Region of interest
